# Long-term trends in incidence and mortality in *Staphylococcus aureus* bacteraemia; a retrospective population-based study from Central Norway 1996–2022

**DOI:** 10.1186/s12879-026-13315-5

**Published:** 2026-04-22

**Authors:** Ingvild Haugan, Helene Marie Flatby, Birgitta Ehrnström, Nina Vibeche Skei, Randi Marie Mohus, Erik Solligård, Frode Width Gran, Lise Tuset Gustad, Stian Lydersen, Christina Gabrielsen Ås, Jan Kristian Damås, Jan Egil Afset

**Affiliations:** 1 Norwegian University of Science and Technology, Central Norway Sepsis Research Centre, Trondheim, Norway; 2 Department of Medical Microbiology, St. Olav’s University Hospital, Trondheim, Norway; 3 Department of Clinical and Molecular Medicine, Norwegian University of Science and Technology, Trondheim, Norway; 4 Department of Infectious Diseases, St. Olav’s University Hospital, Clinic of Medicine, Trondheim, Norway; 5 Department of Intensive Care and Anaesthesia, Nord Trøndelag Hospital Trust, Levanger, Norway; 6 Clinic of Anaesthesia and Intensive Care, St. Olav’s University Hospital, Trondheim, Norway; 7 Department of Circulation and Medical Imaging, Norwegian University of Science and Technology, Trondheim, Norway; 8 Clinic of Laboratory Medicine, St. Olav’s University Hospital, Trondheim, Norway; 9 Faculty of Nursing and Health Sciences, Nord University, Levanger, Norway; 10 Department of Mental Health, Norwegian University of Science and Technology, Trondheim, Norway

**Keywords:** Staphylococcus aureus, Staphylococcus aureus bacteraemia, Epidemiology, Bloodstream infection, Incidence, Mortality, Long-term trends

## Abstract

**Objectives:**

This study aims to examine changes in incidence and mortality in *Staphylococcus aureus* bacteraemia (SAB) over time and between sexes, describe clinical characteristics and asses their associations with 30-day mortality.

**Methods:**

We included 837 first-time SAB episodes from 1996 to 2022 in patients aged 18 years or older recorded in the Nord-Trøndelag Hospital Trust Sepsis Registry. Logistic regression was used to investigate time trends and associations with mortality. The study period was divided into early- (1996–2004), mid- (2005–2013) and late- (2014–2022) periods, and results are reported for each period and overall.

**Results:**

The incidence of SAB increased from 20.0 to 40.3 per 100 000 person years from the early- to the late periods, with males accounting for 522 (62.4%) of all cases. Mortality decreased from 22.6% to 19.0% in males and 29.2% to 20.0% in females. Higher age, malignancy, congestive heart failure, pulmonary infection focus, and unknown focus were associated with increased 30-days mortality.

**Conclusion:**

The incidence rate of SAB increased significantly from 1996 to 2022, with a consistently higher rate in males compared to females. While the highest mortality rate was observed in females in the earliest period, the mortality rate was similar between the sexes in the last period. The decline in mortality occurred despite an ageing patient population and a stable burden of comorbidities. With the exception of age, none of the variables associated with increased or decreased mortality changed significantly over time.

**Clinical trial number:**

Not applicable.

**Supplementary Information:**

The online version contains supplementary material available at 10.1186/s12879-026-13315-5.

## Introduction

*Staphylococcus aureus* has been a recognised cause of bloodstream infections since the naming of *Staphylococcus* in 1880. Knowledge has later accumulated through research and experience, and medical advances have impacted on the characteristics and epidemiology of *S. aureus* infections.

Today, *S. aureus* has become a leading cause of bacteraemia [1, 2], the predominant bacterium detected in osteomyelitis and septic arthritis [3, 4], and the most common cause of infective endocarditis in high income countries (HIC) [5].

The incidence of *S. aureus* bacteraemia (SAB) increased in HIC over the last half of the 20th century, but studies from the late 20th and early 21st centuries report conflicting results on trends [5–7]. Males are at increased risk of SAB compared to females [8] and while they did not investigate incidence, a previous study from Central Norway found that 60.2% of all SAB episodes from 1996 to 2011 occurred in males [9].

Although SAB mortality rate has declined since the pre-antibiotic era, it remains high, with 1 month mortality rates close to 20% in HIC [5, 10]. The previous Central Norway SAB study reported a 30-day mortality rate of 27.3% which remained unchanged over time [9]. Advanced age, comorbidities, infection foci, and female sex may increase SAB mortality [11, 12].

Despite research efforts and gained experience over the 145 years since the naming of *Staphylococcus*, knowledge on SAB remain insufficient to fully understand determinants of high risk and poor outcome. Analyses of long-term population-based data play an important role in the achievement of insight required for optimised management of SAB.

In this study, we analyse temporal trends in the incidence and 30-day mortality of SAB from 1996 to 2022 in males and females in a population-based cohort. We report clinical characteristics across different time periods and evaluate the association between sex, site of acquisition, infection focus, and comorbidities and the 30-day mortality rate.

## Materials and methods

### Setting, population, and definitions

We used clinical data from the Nord-Trøndelag Hospital Trust Sepsis Registry and International Statistical Classification of Diseases and Related Health Problems (ICD)-9 and − 10 codes from patient medical records. The Sepsis Registry was established in 1994 and includes demographic, clinical, and laboratory data collected retrospectively from all episodes of bloodstream infections in patients admitted to either of the two regional hospitals, Namsos and Levanger. These hospitals cover the population of former Nord-Trøndelag County, which had an adult population ranging from 85 716 to 108 327 persons during the study years. Population data was collected from Statistics Norway.

We included all patients ≥ 18 years with an episode of SAB during a hospital admission from 1996 to 2022, provided they had not previously experienced SAB during that period. The onset of an SAB was defined as the time of blood culture sampling. We calculated incidence rates as the number of episodes divided by the number of persons in the hospital catchment area and report the rate as episodes per 100 000 person years. For all sex stratified analyses we used the sex registered by the Sepsis Registry which reported the legal sex of the patient on admission.

For assessment of comorbidities we used the updated Charlson Comorbidity Index (uCCI) by Quan et al. and did not score for age [13, 14]. Sepsis was defined as a Sequential Organ Failure Assessment (SOFA) score of ≥ 2 [15]. We allowed up to two SOFA points for reduced peripheral oxygen saturation in episodes lacking arterial blood gas data as described by Valik et al. [16]. When time of administration was missing, we only scored for vasopressor if mean arterial pressure was < 65 mmHg at the onset of SAB. Missing data were scored zero. We report mortality as all-cause mortality within 30 days after the blood culture was drawn.

Focus of infection was identified using ICD-9 and − 10 codes assigned to each episode. Lower respiratory tract infections other than pyothorax or empyema required radiological findings supporting the ICD-code diagnosis. Urinary tract infections required either a urine sample with leukocyte esterase and growth of *S. aureus* or radiological evidence in addition to the ICD-code. Growth of *S. aureus* from a sterile sample or a definite radiological diagnosis was considered sufficient in the absence of an ICD-code focus. See Supplementary Table 1 for more information on infection foci.

An episode was defined as a hospital-acquired infection if the patient was readmitted with SAB within 48 h after a previous hospital stay, the onset of SAB occurred 48 h or more after the admission time of the current hospital stay, the SAB resulted from surgery or a procedure performed during the hospital stay, or the SAB resulted from surgery performed in hospital within the last 30 days (90 days if deep infection or organ and cavity infections after implant surgery) [17].

### Statistical analysis

We report descriptive statistics as mean for continuous variables and counts and percentages for categorical variables. These will be reported for the total study, as well as separately for three 9-year intervals, referred to as early- (1996–2004), mid- (2005–2013), and late periods (2014–2022).

For trends in incidence rate we used logistic regression with SAB episode as dependent variable and calendar year as main covariate adjusting for age, and we studied the effect of sex on incidence rate by including sex as covariate.

We used logistic regression to analyse 30-day mortality with calendar year as main covariate and adjusted for patient age.

These analyses were also carried out using fractional polynomials to account for possibly nonlinear effects of calendar year. In addition, we performed all the above analyses separately for males and females.

To examine changes over the study years, we used logistic regression with sex, comorbidities, place of acquisition, or focus of infection as dependent variable, and calendar year as main covariate, adjusting for patient age. We replaced calendar year with 30-day mortality in the regression analyses to investigate associations with mortality and adjusted for year in addition to age.

We report 95% confidence intervals (CI) where relevant, and regard p-values under 0.05 to represent statistical significance.

Stata version 19.5 (StataCorp LLC, College Station, TX, USA) was used for all statistical analyses.

### Ethical approval

The study was conducted in accordance with the Declaration of Helsinki and approved by the Regional Committee for Medical and Health Research Ethics, Norway (REK North, 2020/171888) and Nord-Trøndelag Hospital Trust Data Access Committee (2020/8704). We used anonymised registry data which were processed in compliance with the General Data Protection Regulation (GDPR). The Data Protection Impact Assessment (DPIA) was approved by the Head of the Clinic of Laboratory Medicine, St. Olavs hospital.

## Results

### Incidence

The mean annual incidence rate was 31 per 100 000 person years overall, 38 for males, and 23 for females (Table 1). Assuming linearity, the age-adjusted incidence rate increased every year in both the total population, males, and females, with yearly increases of 3.0%, 3.2%, and 2.3%, respectively.

**Table 1:** Person years and incidence rates with odds ratios per year for the increasing incidence

		Total *n* (%)	1996-2004 *n* (%)	2005-2013 *n* (%)	2014-2022 *n* (%)	OR (95% CI) *p*-value
**Person years**		**2 739 833**	**868 315**	**909 215**	**962 303**	
	Male	1 366 489	431 085	452 388	483 016	
	Female	1 373 344	437 230	456 827	479 287	
**Episodes**		**837**	**174**	**275**	**388**	
	Male	522 (62.4)	102 (58.6)	172 (62.6)	248 (63.9)	
	Female	315 (37.6)	72 (41.4)	103 (37.5)	140 (36.1)	
**Incidence rate**		**30.6**	**20.0**	**30.3**	**40.3**	**1.030 (1.0211 to 1.039) < 0.001**
	Male	38.2	23.7	38.0	51.3	1.032 (1.021 to 1.045) < 0.001
	Female	22.9	16.0	23.0	29.0	1.023 (1.008 to 1.038) 0.002

**Fig. 1:**
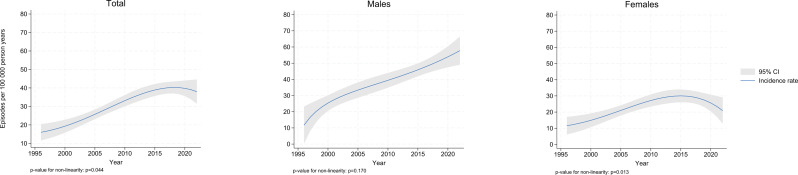
Non-linear age-adjusted model of SAB incidence rate in the total population, males, and females

Analyses with fractional polynomials revealed a non-linear trend in incidence for the total population (*p* = 0.044) and for females (*p* = 0.013), with a decline observed in the most recent study years (Fig. 1).

### Episodes, sex, and age

A total of 837 episodes of SAB were registered from 1996 to 2022 with 522 (62.4%) occurring in males (Table 1). We retrieved clinical data for all but one episode in the mid period and ICD-codes for all except one episode in the early period (Table 2).

**Table 2:** Patient characteristics and mortality with odds ratios for prevalence change per year

		Total *n* (%)	1996-2004 *n* (%)	2005-2013 *n* (%)	2014-2022 *n* (%)	OR (95% CI) *p*-value
**Episodes**		837	174	275	388	
Age, mean		71.6	69.3	71.6	72.6	
	Male	70.9	69.7	70.0	72.0	
	Female	72.9	68.8	74.6	73.8	
Age ≥ 65		609 (72.8)	118 (67.8)	197 (71.6)	294 (75.8)	1.023 (1.002 to 1.045) 0.029
Comorbidities*						
uCCI^#^, mean		1.7	1.5	2.1	1.5	
uCCI^#^ > 0		515 (61.6)	102 (58.6)	177 (64.6)	236 (60.8)	1.000 (0.981 to 1.020) 0.983
Diabetes		185 (22.1)	24 (13.8)	63 (23.0)	98 (25.7)	1.033 (1.009 to 1.057) 0.008
Malignancy		175 (20.9)	35 (20.1)	66 (24.1)	74 (19.1)	0.993 (0.971 to 1.017) 0.572
Congestive heart failure		111 (13.3)	13 (7.5)	45 (16.4)	53 (13.7)	1.024 (0.994 to 1.055) 0.114
Heart valve disease		68 (8.1)	11 (6.3)	19 (6.9)	38 (9.8)	1.028 (0.991 to 1.067) 0.141
Dementia		61 (7.3)	13 (7.5)	18 (6.6)	30 (7.7)	0.994 (0.956 to 1.034) 0.752
Chronic kidney disease		61 (7.3)	9 (5.2)	20 (7.3)	32 (8.3)	1.019 (0.983 to 1.058) 0.307
Haemodialysis		43 (5.1)	7 (4.0)	13 (4.7)	23 (5.9)	1.037 (0.991 to 1.085) 0.119
Liver disease		19 (2.3)	2 (1.2)	10 (3.7)	7 (1.8)	1.007 (0.947 to 1.071) 0.831
Acquisition*						
Hospital-acquired infection		255 (30.5)	69 (39.7)	87 (31.6)	99 (25.5)	0.967 (0.947 to 0.987) 0.001
**Severity at onset**,** ICU**, echocardiography*						
SOFA^#^ score ≥ 2		496 (59.3)	95 (54.6)	159 (58.0)	242 (62.4)	1.013 (0.993 to 1.032) 0.208
Intensive care unit admision		126 (15.1)	23 (13.2)	39 (14.2)	64 (16.5)	1.015 (0.988 to 1.042) 0.273
Echocardiography		333 (39.8)	47 (27.0)	83 (30.3)	203 (52.3)	1.079 (1.056 to 1.102) < 0.001
**Focus of infection^**						
Primary SAB^#^/unknown focus		338 (40.4)	75 (43.4)	106 (38.6)	157 (40.5)	0.993 (0.974 to 1.013) 0.490
Pneumonia		100 (12.0)	21 (12.1)	30 (10.9)	38 (9.8)	0.977 (0.949 to 1.005) 0.105
Foreign body infection		91 (10.9)	18 (10.4)	27 (9.8)	46 (11.9)	1.015 (0.984 to 1.047) 0.340
Skin and soft tissue infection		85 (10.2)	13 (7.5)	31 (11.3)	41 (10.6)	1.019 (0.987 to 1.052) 0.251
Septic arthritis		55 (6.6)	9 (5.2)	15 (5.5)	31 (8.0)	1.016 (0.977 to 1.057) 0.416
Osteomyelitis, spondylodiscitis		63 (7.5)	13 (7.5)	14 (5.1)	36 (9.3)	1.011 (0.975 to 1.047) 0.565
Infective endocarditis		39 (4.7)	5 (2.9)	9 (3.3)	25 (6.4)	1.065 (1.013 to 1.119) 0.014
**30-day mortality**		187 (22.3)	44 (25.3)	68 (24.7)	75 (19.3)	0.972 (0.950 to 0.995) 0.018
	Male	110 (21.1)	23 (22.6)	40 (23.3)	47 (19.0)	0.983 (0.955 to 1.013) 0.268
	Female	77 (24.4)	21 (29.2)	28 (27.2)	28 (20.0)	0.955 (0.920 to 0.992) 0.019

*Missing data from one episode from the mid time period

^Missing data from one episode from the first time period

**Fig. 2:**
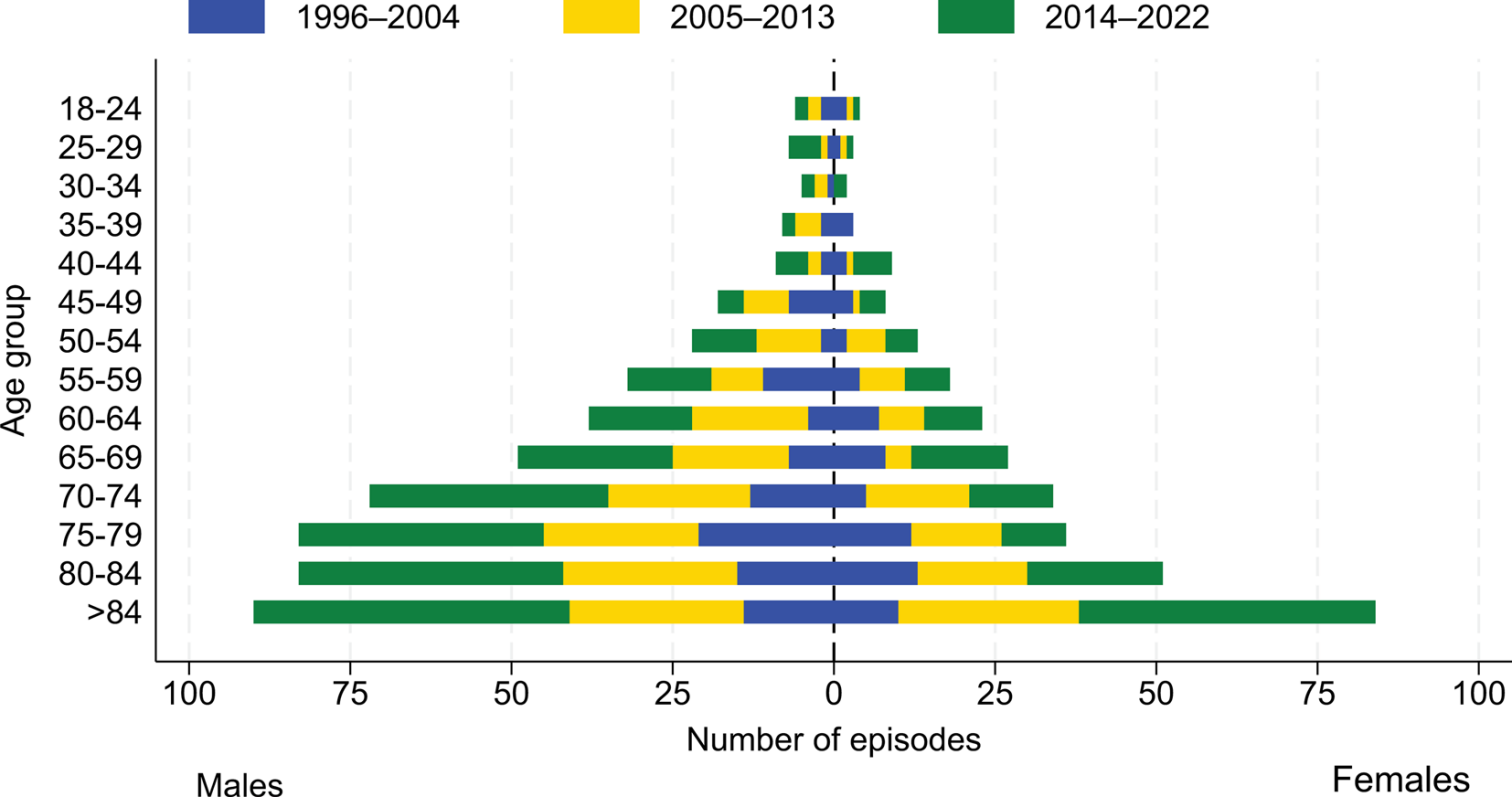
Age group proportion by sex, colour coded for period

^#^uCCI = updated Charlsons Comorbidity Index, SOFA = Sequential Organ Failure Assessment, SAB = *Staphylococcus aureus* bacteraemia Patient age ranged from 18 to 98 years, and the proportion of patients ≥ 65 years increased significantly over time (Fig. 2; Table 2)

### Comorbidities

A total of 515 (62%) patients had a uCCI score > 0, and this proportion remained stable throughout the study period (Table 2). The most common comorbidities were diabetes and malignancy. The proportion of patients with diabetes increased, and we observed non-significant upwards trends in congestive heart failure, heart valve disease, chronic kidney disease, and haemodialysis.

### Acquisition

We classified the SAB as hospital-acquired in 255 (31%) patients. The proportion of infections acquired in hospital declined significantly from the early to the late period (Table 2).

### Severity

At onset of infection, 344 (41%) patients had sepsis, defined as SOFA score ≥ 2, and 126 (16%) were admitted to an intensive care unit (Table 2). Neither of these proportions changed significantly over the study period.

### Infection focus

After primary SAB or unknown focus (*n* = 338, 40%), the most common foci were lungs (*n* = 100, 12%), foreign body (*n* = 91, 11%), and skin and soft tissue (*n* = 85, 10%) (Table 2). Patients diagnosed with infective endocarditis increased from 5 (2.9%) in the early period to 25 (6.4%) in the late. The proportion of patients examined with echocardiography increased significantly.

### Mortality

The all-cause 30-day age-adjusted mortality rate was 22% in the total study cohort, 21% in males, and 24% in females (Table 1). Assuming linearity, the age-adjusted odds for 30-day mortality decreased with 2.8% per year. In sex stratified analyses, this decrease was only significant in females (Table 2). Analyses with fractional polynomials did not show significant deviation from linearity.

### Associations with mortality

**Fig. 3:**
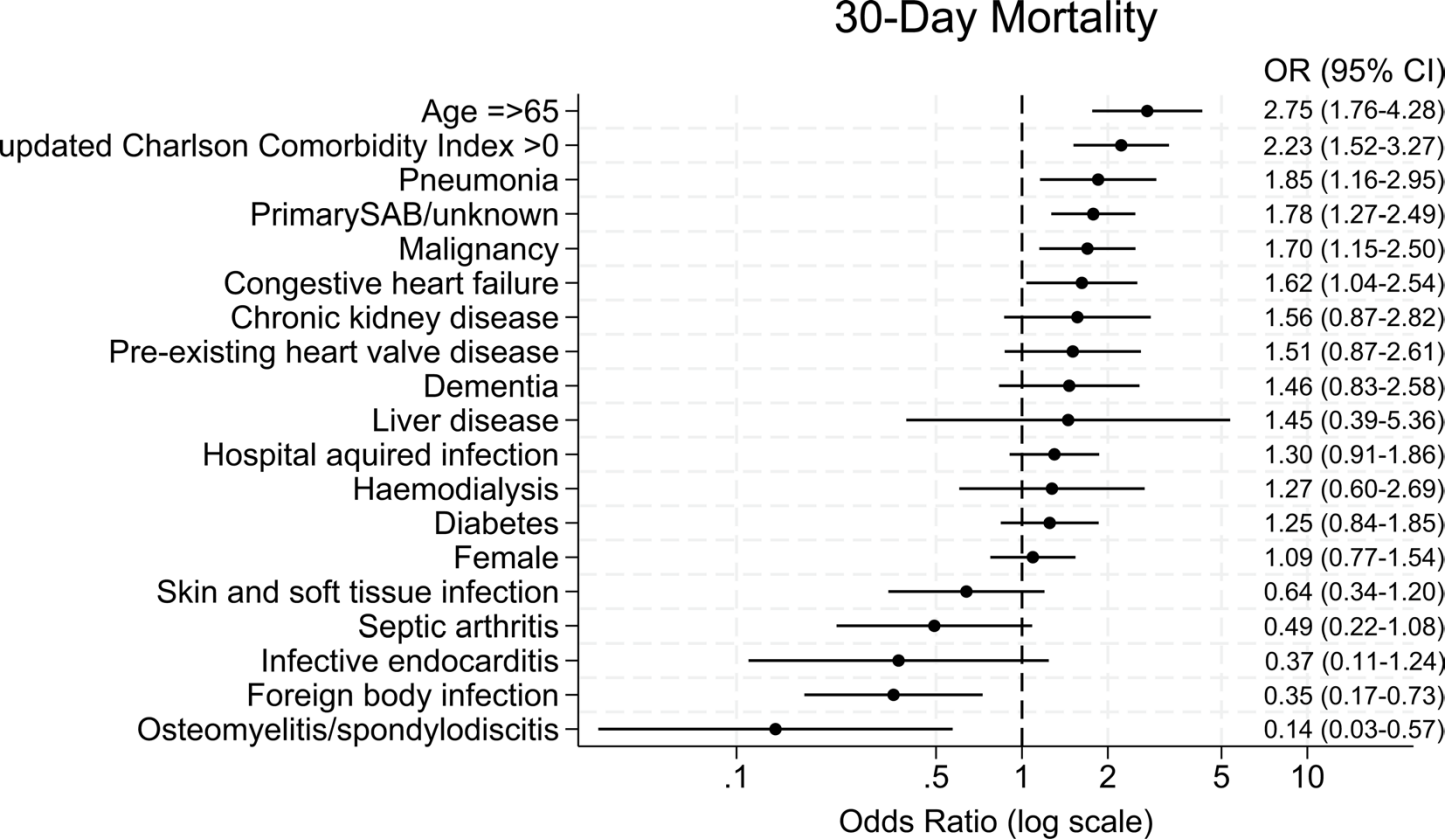
Odds ratios for 30-day mortality with 95% confidence intervals. Analysis of Age ≥ 65 is adjusted for sex and year, analysis of Female is adjusted for age and year, and all other analyses are adjusted for age, sex, and year

Among demographic variables, age ≥ 65 years was associated with a 2.75 increase in the odds of 30-day mortality compared to younger patients, after adjusting for sex and calendar year (Fig. 3).

The presence of a relevant comorbidities, indicated by a uCCI score of > 0, was associated with 2.23 higher odds for 30-day mortality compared to those with a score of zero after adjusting for sex, age, and calendar year. Those with pre-existing malignancy or congestive heart failure had significantly higher adjusted 30-day mortality rates than those without.

Among infection foci, lungs were associated with the highest 30-day mortality followed by those with primary SAB or unknown focus. Having a foreign body infection or osteomyelitis/spondylodiscitis were associated with reduced odds for 30-day mortality. The trends towards lower 30-days mortality rates in skin and soft tissue infections, septic arthritis, and endocarditis, were non-significant.

## Discussion

This study over 27 years provides one of the longest population-based analyses of SAB in a high-income setting and found increasing incidence, reduced 30-day mortality, declining proportion of hospital-acquired infections, and rising proportion of endocarditis and use of echocardiography.

The overall SAB incidence rate was 31 per 100 000 person years and increased from 20 in the early to 40 in the late period, a time span in which blood culture sampling more than doubled [18, 19]. Other HIC saw a similar increase in SAB [5, 20] and proposed explanations include an ageing population, increased prevalence of chronic diseases, and increased use of medical devices and invasive procedures. In our study, patient age and diabetes mellitus, both associated with SAB, increased over time [5, 21].

Our nonlinear models show that the incidence rate increased only in males in the last years. The final three years of the study coincide with the first years of the Coronavirus Disease of 2019 (COVID-19) pandemic. A systematic review from 2021 concluded that *S. aureus* was the most common organism in co- and secondary infections in bloodstream and lungs in patients with COVID-19 [22]. Further research is required to investigate if the incidence increase in males at the end of our study is related to the COVID-19 pandemic. The decreasing SAB incidence rate observed in females towards the end of the study started prior to the COVID-19 pandemic and cannot be fully explained by a general decrease in communicable diseases due to implementation of infection control measures during the pandemic.

The SAB incidence remained higher in males than females throughout the study period. Several studies have found a higher risk for bloodstream infection with *S. aureus* in males than females [5, 8], however the reasons for this difference remain unexplained. Carriage of *S. aureus* is a potential risk factor for SAB, and the prevalence of *S. aureus* colonisation is higher in males than females [23, 24]. Sex hormone status is suggested as one explanation for these differences, and testosterone levels have been linked to *S. aureus* colonisation [25, 26]. One study found that social and behavioural factors together with comorbid conditions explained 34% of the difference in risk of bloodstream infections between sexes [8]. Sex and age have been associated with different *S. aureus spa*-types in carriers [27] and certain clonal complexes with invasive disease [28]. Conclusively, both human biological and behavioural factors as well as bacterial characteristics may influence sex differences in *S. aureus* carriage and SAB.

The overall 30-day mortality rate in our study was 22% and declined over time, which is similar to results from a recent systematic review, but lower than the previous Central Norway study [9, 10]. This occurred despite population ageing and a stable burden of comorbidities. Females are reported to have higher SAB mortality risk than males [11]. As shown in Table 2, the mortality rate in our study was higher in females than males in the early period and similar between the sexes in the late period. Statistical power is reduced in sex-stratified analyses and further investigations are required to assess if this observation represents a true difference in the temporal change in mortality rate in females and males.

Coinciding with our study period, the international medical community introduced scoring tools and initiated the Surviving Sepsis Campaign (SCC) to improve outcomes in sepsis patients [15, 29]. The use of echocardiography and radiological investigations were increasingly implemented as standard of care in patients with SAB [30]. Establishing a causal relationship between implementation of guidelines and mortality is inherently challenging. Previous studies have reported decreasing mortality rates associated with compliance to SCC guidelines, and reduced mortality among SAB patients who received infectious disease consultation and were examined with echocardiography [31–34]. We did not investigate the impacts of such interventions on mortality in this study.

We found associations with higher SAB mortality in pneumonia and primary SAB or unknown focus, which is in concordance with previous studies [5, 12]. Unlike prior reports we observed a surprising trend towards reduced mortality in infective endocarditis. More patients were investigated with echocardiography in the late- compared to the early period, and the proportion of patients diagnosed with infective endocarditis increased. The low mortality rate in infective endocarditis may reflect the overall declining mortality. It is possible that the group of patients with pneumonia and primary SAB or unknown focus included some unrecognised deep and complicated infections reflecting less intensive medical management and more limited diagnostic work up compared to the endocarditis group. Both the presence of medically complicating factors, severe acute illness, and an assumed poor prognosis, could influence medical decision-making as well as the overall outcome of SAB. If patients in the endocarditis group more often received a correct diagnosis, this could be associated with improved outcomes if it resulted in more appropriate management than in patients with missed focus. Whether foci of infection affect mortality directly, indirectly, and/or act as markers for other factors which impact the outcome should be addressed in future studies.

One of the limitations of this study is the retrospective data collection, which is vulnerable to information bias. Furthermore, the validity of ICD-codes to determine infection focus may be influenced by changing ICD coding practices over time and that the purpose of such coding is not solely medical. A notable strength of our study is that it presents results from a population-based cohort in a restricted geographical area with a homogenous population over nearly three decades, which allows for reliable analyses of time trends. On the other hand, the observations made in this study may not be generalisable to other populations. While SAB is a heterogeneous disease entity, and subgroup analyses would have been relevant, the limited size of several subgroups restricted the statistical power to perform subgroup analyses.

Over the course of the 27-year time period, it is likely that developments in healthcare practice, diagnostics, and population dynamics not included in this study have occurred and influenced both the epidemiology and outcome in SAB. We did not have data on blood culture sampling practices in our study population and therefore cannot determine to which degree changes in sampling rate impacted on the measured incidence rate and observed mortality rate. However, Danielsen et al. [19] found only a slight decrease in the blood culture positivity rate despite a marked increasing sampling rate in Norway, supporting a true increase in bloodstream infections overall.

## Conclusion

In this population-based cohort study spanning over 27 years, SAB incidence is still increasing and remains higher in males than females. The 30-day mortality rate stayed high, but we did observe a decrease in mortality over time that was significant in females but not in males. Sepsis management and identification of infectious foci may have improved concurrently with this decline. Further insight into the pathogenesis and sex differences of invasive *S. aureus* infection is required in order to personalise treatment and identify patients at risk of complicated infections and adverse outcome.

## Supplementary Information

Below is the link to the electronic supplementary material.

Supplementary Material 1

## Data Availability

The majority of data that supports the findings of this study are available from the Nord-Trøndelag Hospital Trust Sepsis Registry, but restrictions apply to the availability of these data, which were used under license for the current study and so are not publicly available. The data are available upon request for research projects after approval from the local Data Access Committee.

## Abbreviations

SAB: *Staphylococcus aureus* bacteraemia
HIC: High income countries
ICD: International Statistical Classification of Diseases and Related Health Problems
uCCI: Updated Charlson Comorbidity Index
SOFA: Sequential Organ Failure Assessment
CI: Confidence Interval
OR: Odds Ratio
DPIA: Data Protection Impact Assessment
GDPR: General Data Protection Regulation
