# Supplementary material for: Long-term trends in incidence and mortality in Staphylococcus aureus bacteraemia; a retrospective population-based study from Central Norway 1996–2022
Source: BMC Infect Dis. 2026 Apr 22;26:1081. doi: 10.1186/s12879-026-13315-5 (PMC13235103; doi:10.1186/s12879-026-13315-5)
Supplement: Supplementary file 1 — Supplementary Material 1 [file 12879_2026_13315_MOESM1_ESM.docx]

| **Infection focus in paper*** | **Subtype** | **Number** | **Criteria fulfilled** | **Number** |
| --- | --- | --- | --- | --- |
| Primary SAB or unknown focus |  |  |  |  |
|  | Primary SAB or unknown focus | 251 | No ICD code for infection focus, no growth of S. aureus from deep or sterile sample, no radiological evidence | 251 |
|  | Uncertain focus | 87 | ICD code for pneumonia, infectious COPD exacerbation, acute bronchitis or unspecified infection in lower respiratory tract without radiologial or microbiological support | 39 |
|  |  |  | ICD code for tonsillitis or unspecified upper airway infection without radiological or microbiological support of abscess or sinusitis | 2 |
|  |  |  | ICD code for UTI without S. aureus and leukocytes in urine | 46 |
| Pneumonia |  |  |  |  |
|  | Pneumonia | 86 | ICD code for pneumonia or pneumonitis AND radiological evidence or growth of S. aureus from lower respiratory material | 85 |
|  |  |  | No ICD code for lower respiratory tract infection but evidence on CT scan | 1 |
|  | Empyema or lung abscess with or without pneumonia | 14 | ICD code for empyema, pyothorax or abscess | 13 |
|  |  |  | No ICD code for lower respiratory tract infection but growth of S. aureus from pleural fluid | 1 |
| Foreign body infections |  |  |  |  |
|  | Prosthetic endocarditis including pacemaker infection | 14 | ICD code for (aute/subacute/unspecified) endocarditis AND registered presence of prosthetic heart valve | 9 |
|  |  |  | ICD code for infection and inflammatory reaction due to cardiac valve prosthesis | 2 |
|  |  |  | ICD code for (acute/subacute/unspecified) endocarditis AND radioloical evidence of pacemaker infection | 3 |
|  | Vascular graft infection | 21 | ICD code for infection and inflammatory reaction due to other cardiac and vascular devices, implants and grafts NOT fulfilling criteria for prosethtic endocarditis including pacemaker infection | 20 |
|  |  |  | ICD code for unspecified foreign body infection and registered information on infected vascular graft NOT fulfilling criteria for prosethtic endocarditis including pacemaker infection | 1 |
|  | Prosthetic arthritis | 38 | ICD code for infection and inflammatory reaction due to internal joint prosthesis | 32 |
|  |  |  | ICD code for infection and inflammatory reaction due to internal fixation device or other internal orthopaedic prosthetic devices, implants and grafts AND registered joint prosthesis or growth of S. aureus from joint aspirate | 2 |
|  |  |  | No ICD code for arthritis but registered joint prosthesis AND growth of S. aureus from joint aspirate | 1 |
|  |  |  | No ICD code for arthritis but registered joint prosthesis AND radiological evidence or growth of S. aureus from joint aspirate or tissue sample from joint | 3 |
|  | Other orthopaedic foreign body infection | 15 | ICD code for infection and inflammatory reaction due to internal fixation device or other internal orthopaedic prosthetic devices, implants and grafts NOT fulfilling criteria for prosthetic arthritis | 14 |
|  |  |  | No ICD code for foreign body infection but registered orthopaedic foreign body AND growth of S. aureus from relevant sample NOT fulfilling criteria for prosthetic arthritis | 1 |
|  | Infection in unspecified foreign body | 8 | ICD code for infection and inflammatory reaction due to an unspecified device implant and graft or other internal prosthetic devices, implants and grafts NOT fulfilling criteria for prosthetic endocarditis including pacemaker infection or prosthetic arthritis | 8 |
| Skin and soft tissue infections |  |  |  |  |
|  | All SSTI | 85 | ICD code for erysipelas, cellulitis, cutaneous abscess, pyoderma, gas gangrene, local infections of skin and subcutaneous tissue, infective myositis, mediastinal abscess, abscess of tendon sheath, bursitis, abscess of bursa or infection of amputation stump | 80 |
|  |  |  | No ICD code for SSTI but growth of S. aureus in superficial skin or soft tissue sample | 5 |
| Septic arthritis |  |  |  |  |
|  | Native endocarditis | 55 | ICD code for Staphylococcal arthritis or pyogenitc arthritis unspecified AND no orthopaedic foreign body in joint | 41 |
|  |  |  | No ICD code for arthritis but growth of S. aureus from joint aspirate or tissue sample from joint and no orthopaedic foreign body in joint | 11 |
|  |  |  | ICD code for arthritis dut to other specified bacerial agents AND ICD code for Staphylococcus aureus as the cause of disease classified to other chapter and no orthopaedic foreign body in joint | 3 |
| Osteomyelitis, spondylodiscitis |  |  |  |  |
|  | Osteomyelitis | 30 | ICD code for osteomyelitis, acute haemotogenous osteomyelitis, other acute osteomyelitis, subacute osteomyelitis, chronic osteomyelitis with draining sinus, other chronic osteomyelitis, other and unspecified osteomyelitis | 27 |
|  |  |  | No ICD code for osteomyelitis but radiological evidence | 2 |
|  |  |  | No ICD code for osteomyelitis but growth of S. aureus from tissue sample | 1 |
|  | Spondylodiscitis | 36 | ICD code for osteomyelitis of vertebra, infection of intervertebral disc or other infective spondylopathies | 28 |
|  |  |  | ICD code for intraspinal or extra- or subdural abscess | 7 |
|  |  |  | ICD code for other and unspecific disk disorder and ICD code for osteomyelitis | 1 |
| Infective endocarditis |  |  |  |  |
|  | Native infective endocarditis | 39 | ICD code for acute and subacute or unspecified endocarditis and no evidence of prosthetic heart valve | 39 |
|  |  |  |  |  |
|  |  |  |  |  |
| * Some episodes have multiple foci | |  |  |  |
